# Novel microwell assay with high throughput and minimum consumption for organic solvents in the charge transfer-based spectrophotometric determination of clarithromycin in pharmaceutical formulations

**DOI:** 10.1186/1752-153X-7-172

**Published:** 2013-10-31

**Authors:** Ibrahim A Darwish, Mohammed A Alqarni, Tanveer A Wani

**Affiliations:** 1 Department of Pharmaceutical Chemistry, College of Pharmacy, King Saud University, P.O. Box 2457, Riyadh 11451, Saudi Arabia

**Keywords:** Clarithromycin, Microwell spectrophotometric assay, Charge-transfer, Organic solvents, High analysis throughput

## Abstract

**Background:**

Clarithromycin (CLM) is a semi-synthetic macrolide antibiotic with a broad antibacterial spectrum. It has a potent activity against *Myc. Pneumonia*, *Legionella Spp.*, *H. Influenza*, and *Mor. Catarrhalis*. It is also used for prevention and treatment of disseminated *M. Avium* infections in patients with AIDS. The therapeutic importance and wide use of CLM promotes the growing interest in developing proper methods for its determination in bulk and pharmaceutical formulations.

**Results:**

The present study describes the development and validation of a novel assay that can increase the throughput and reduce the consumption of organic solvents in the charge transfer (CT)-based spectrophotometric determination of CLM. In this assay, the CT reaction between CLM as n-electron donor and 2,3-dichloro-5,6-dicyano-1,4-benzoquinone (DDQ) as a π-electron acceptor was performed in the 96-microwells of an assay plate. The color signals of the CT complex were measured at 450 nm by microwell-plate absorbance reader. The linear range of the assay was 20−850 μg mL^−1^. The limits of detection and quantitation were 15.5 and 51.2 μg mL^−1^, respectively. The proposed assay gave very high precisions; the relative standard deviation (RSD) values did not exceed 1.82%.

**Conclusions:**

The assay described herein has a high throughput property that facilitates the processing of large number of samples in a reasonable time. As well, it consumes minimum volumes of organic solvents, thus it significantly reduces the exposures of the analysts to the toxic effects of organic solvents, and reduce the analysis cost by 50-folds. The results demonstrated that the proposed assay has great practical value in the routine analysis of CLM in quality control laboratories.

## Background

**Figure 1:**
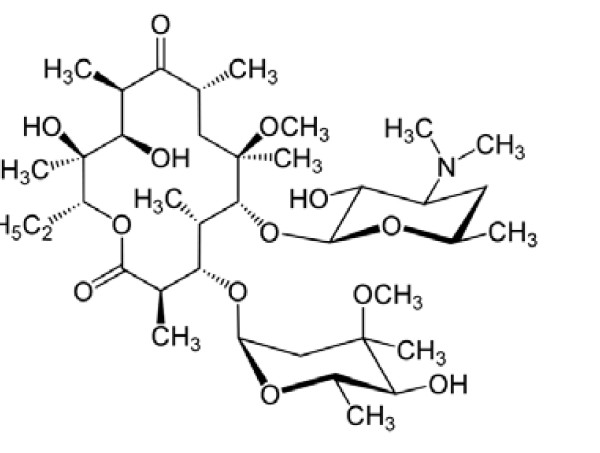
The chemical structure of clarithromycin (CLM).

Clarithromycin (CLM), 6-*O*-methylerythromycin (Figure 1) is a semi-synthetic macrolide antibiotic with a broad antibacterial spectrum. It has a potent activity against *Myc. Pneumonia*, *Legionella Spp.*, *H. Influenza*, and *Mor. Catarrhalis*[1,2]. It is also used for prevention and treatment of disseminated *M. Avium* infections in patients with AIDS [1]. CLM interferes with RNA dependent bacterial protein synthesis, resulting in a bateriostatic effect on pathogens [2].

The therapeutic importance of CLM was behind the growing interest in the development of analytical methods for its determination in raw material, pharmaceutical formulations and/or biological fluids. A literature survey revealed that most of the reported methods for determination of CLM are HPLC with electrochemical [3,4] or mass spectrometric detector [5-7]. These methods were devoted to the determination of CLM in biological fluids and employed sophisticated and expensive instrumentation that are mostly not available in pharmaceutical quality control laboratories. Therefore, the applications of these methods for determination of CLM in pharmaceutical formulations are limited.

Spectrophotometry is the most widely used technique in pharmaceutical analysis because of its inherent simplicity and wide availability in most quality control laboratories [8]. CLM molecule lacks a suitable chromophore that enables its determination in the pharmaceutical formulations based on the direct measurement of its UV absorption. Therefore, derivatization of CLM was necessary for its spectrophotometric determination. Unfortunately, most the spectrophotometric methods reported for the determination of CLM in its pharmaceutical formulations [8-17] were associated with some major drawbacks. These drawbacks included tedious liquid-liquid extraction procedures using large volumes of organic solvents in the methods based on formation of extractive ion-pair associates [11-15], and the employment of multiple-steps and long time for completing the whole procedure [16,17]. Therefore, the development of a new alternative spectrophotometric method for determination of CLM in its pharmaceutical formulations is very essential.

The charge-transfer (CT) reactions are widely employed in the development of simple and convenient spectrophotometric methods for many pharmaceutical compounds [18-23]. Few CT-based spectrophotometric methods have been described for determination of CLM [24,25]. These methods employed the conventional non-automated technique and consequently their throughput is low, and their applications in pharmaceutical quality control laboratories are limited. Moreover, these methods consumed large volumes of organic solvents, which leads to high analysis cost, and more importantly, the incidence of exposure of the analysts to the toxic effects of the organic solvents [26-30] such as neurotoxic symptoms [26], increased risk of some reproductive outcomes among women [27], increased risk of spontaneous abortion among pregnant women [28,29], and increased risk of lymphohaemalopoietic cancer (leukemia and lymphomas) in both men and women [30]. Reduction of human exposure to organic solvents is one of the main objectives of hygienists, public authorities, World Health Organization, environment protection agencies, and occupational safety and health administrations. For these reasons, investigating new alternative methodology to reduce the consumption of organic solvents in CT-based spectrophotometric analysis of CLM is very important.

The present study describes the development and validation of a novel CT-based spectrophotometric assay for determination of CLM in pharmaceutical formulations. In this assay, the reaction was carried out in 96-microwell assay plates and the color signals were measured by microwell-plate reader. The assay described herein offered three major advantages: (1) providing a high throughput analytical methodology that can facilitate the processing of large number of samples in a relatively short time, (2) reduction in the exposures of the analysts to the toxic effects of organic solvents, thus it leads to reduction in the analysis cost by 50-folds.

## Experimental

### Apparatus

Microwell-plate absorbance reader (ELx 808, Bio-Tek Instruments Inc. Winooski, USA) was used for all the measurements in 96-microwell plates. UV-1601 PC (Shimadzu, Kyoto, Japan) ultraviolet-visible spectrophotometer with matched 1 cm quartz cells was used for recording the absorption spectra. 96−Microwell plates were a product of Corning/Costar Inc. (Cambridge, USA). Finnpipette adjustable 8−channel-pipette was obtained from Sigma Chemical Co. (St. Louis, MO, USA).

### Chemicals and pharmaceutical formulations

Secondary standard clarithromycin (CLM) was obtained from Riyadh Pharma Industries (Riyadh, Saudi Arabia); purity was 99.96%. DDQ (Merck, Germany) was 1 mg/mL in methanol and it was prepared fresh daily. Klaricid® tablets (Abbot Industries, Italy) labeled to contain 250 mg CLM were obtained from the local market.

### Preparation of standard solutions

Into a 10-mL calibrated flask, 20 mg of CLM was accurately weighed, dissolved in 5 mL methanol, and completed to volume with the same solvent. This stock solution was diluted with methanol to obtain the suitable concentrations in the linear range of the assay.

### Preparation of tablet sample solutions

Twenty tablets were weighed and finely powdered. A quantity of the powder equivalent to 20 mg of CLM was transferred into a 10-mL calibrated flask, dissolved in 5 mL methanol, swirled and sonicated for 5 min, completed to volume with the methanol, shaken well for 15 min, and filtered. The first portion of the filtrate was rejected, and a measured volume of the filtrate was diluted quantitatively with methanol to yield the suitable concentrations in the linear range of the assay.

### General analytical procedure

Accurately measured aliquots (100 μL) of the standard or sample solution of varying concentrations of CLM (40–1700 μg mL^−1^) were transferred into wells of 96-microwell assay plates. One hundred microliters of DDQ solution (1 mg/mL) was added, and the reaction was allowed to proceed at room temperature (25 ± 1°C) for 5 min. The absorbances of the resulting solutions were measured at 450 nm by the microwell-plate reader. Blank wells were treated similarly except 100 μL of methanol was used instead of sample, and the absorbances of the blank wells were subtracted from those of the other wells.

### Determination of molar ratio

The Job’s method of continuous variation [31] was employed. Master equimolar solutions (2×10^─3^ M) of each of CLM and DDQ were prepared. Series of 200 μL portions of the master solutions of CLM with DDQ were made up comprising different complementary ratios (0:10, 1:9, ……, 9:1, 10:0, inclusive) in each well of the assay plate. The reaction was allowed to proceed at room temperature (25 ± 1°C) for 5 min. The absorbances of the developed colors were measured at 450 nm by the microwell-plate reader against blank wells treated similarly except methanol was used instead of CLM sample. The measured absorbances were plotted as a function of CLM mole fraction. The generated plot was used for determination the molar ration of CLM:DDQ.

## Results and discussion

### Strategy for assay development

CLM was selected for this study based on its therapeutic importance and the urgent need for a new spectrophotometric methodology that can overcome the major drawback of the existing methods for its determination. The structure of the CLM contains a macrocyclic lactone and a neutral sugar moiety attached to the lactone (Figure 1). Another important structural characteristic is the presence of other sugar moiety containing a dimethylamine group, which confers to the CLM molecule a basic behavior and makes CLM a potential n-electron donating substances [32]. Based on this fact, CT reaction with electron acceptor was considered in this study.

Previous studies for the CT reactions of polyhalo-/polycyanoquinones electron acceptors revealed that DDQ was one of the most reactive reagents, as its CT reaction proceeds rapidly, and yielded high sensitive assays when compared with other polyhaloquinones [23]. For these reasons, DDQ was selected as electron acceptor in the development of the assay described herein.

The following sections describe the establishment of the optimization factors that influence the chemical reaction and the analytical performance of the proposed assay.

### Absorption spectral characteristics

**Figure 2:**
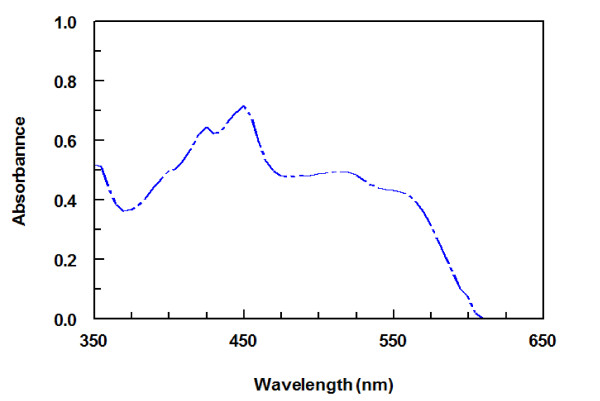
**Absorption spectrum of reaction product of CLM (100 μg mL**^**−1**^**) with DDQ (1 mg mL**^**−1**^**).**

The interaction of CLM with DDQ was allowed to proceed at room temperature, and the absorption spectrum of the produced chromogen was recorded. CLM gave red colored chromogen showing absorption maximum at 450 nm (Figure 2). This band was attributed to the formation of the radical anion DDQ^-^[33], which was probably formed by the dissociation of an original donor-acceptor (D-A) complex:

$$\begin{array}{l} D+A⇌\left(D‐A\right)⇌\begin{array}{c} \mathrm{Polar}\\ \mathrm{solvent} \end{array}D^{+}+A^{‐}\\ \;\text{complex}\;\text{radical}\;\mathrm{ions} \end{array}$$

Further support of this assignment was provided by the absorption maxima with those of DDQ radical anion produced by the iodide reduction assay [33]. The dissociation of the (D-A) complex was promoted by the high ionizing power of the polar solvent and the resulting peaks in the absorption spectra of CLM-DDQ reaction mixtures were similar to the maxima of the radical anions of the acceptors obtained by the iodide reduction assay [33].

### Optimization of experimental conditions

The optimization of experimental conditions affecting the reaction in the 96-well format was investigated by altering each reaction variable in a turn while keeping the others constant. In all cases, measurements were carried out at 450 nm by a microwell plate reader.

#### Effect of solvent

The first factor evaluated in this study was the solvent employed. The solvent plays an important role in CT reactions, since it must be able to facilitate the total CT and then allow the complex dissociation and stabilization of the radical anion formed, which is the absorbing molecule. Previous study [23] demonstrated that solvents with high dielectric constant are more effective to execute this task. Taking this fact into account, the reaction was tested in different solvents (methanol, ethanol, propanol, butanol, acetone, and acetonitrile). Although the highest dielectric constant of acetonitrile; however, the best sensitivity was achieved when methanol was used. This was probably attributed to the ability of methanol to form stable hydrogen bonds with the radical anion. Therefore, methanol was used in all further experiments.

#### Effect of DDQ concentration

**Figure 3:**
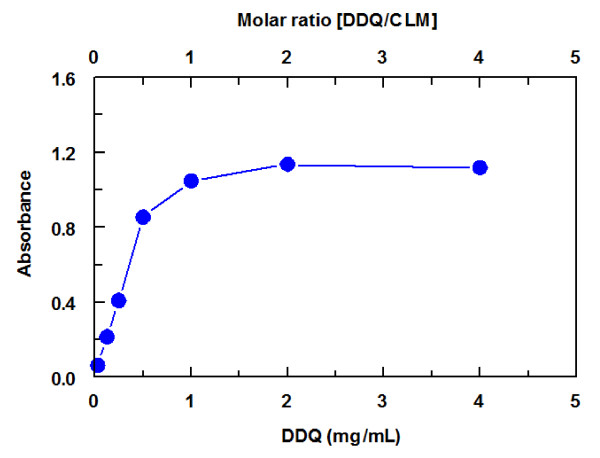
Effect of DDQ concentration on its reaction with CLM.

In the chemical derivatization-based spectrophotometric analysis, the maximum conversion of the analyte into absorbing specie depends on the amount of the reagent available in the reaction solution and the equilibrium involved. The effect of DDQ concentration on its reaction with CLM was studied using 100 μL of varying DDQ concentrations (0−4 mg mL^−1^) and 100 μL of a fixed concentration of CLM (650 μg mL^−1^). The final concentrations DDQ and CLM in each reaction well were 0−2 mg mL^−1^ and 325 μg mL^−1^, respectively. The results (Figure 3) indicated that 100 μL of 1 mg mL−1 was the optimum DDQ concentration, as this concentration gave the highest absorbances. We selected this higher concentration for carrying out the reaction for two reasons: (1) to offer enough DDQ concentration for reaction with high concentrations of CLM, when any, in the routine practical application, and (2) higher DDQ concentration offers, in accordance, high precise readings. As shown in Figure 3, when the DDQ molar concentration was similar to that of the CLM concentration there was a total consumption of CLM, indicating that one mole of CLM reacts with one mole of DDQ reagent.

#### Effect of time and temperature

The optimum reaction time was determined by monitoring the color development in the microwells at room temperature (25 ± 1°C). Complete color development was attained instantaneously, however for higher precision readings, the reaction was allowed to proceed for 5 min. Higher temperatures (40−60°C) had no significant effect on the reaction, thus all investigations were carried out at room temperature. The developed color remained stable at room temperature for at least a further 30 min.

A summary for the optimum conditions is given in Table 1.

**Table 1:** Optimum conditions for the charge-transfer reaction of CLM with DDQ

**Condition**	**Studied range**	**Optimum**
DDQ conc. (mg mL^−1^)	0 − 4	1
Solvent	Different^a^	Methanol
Reaction time (min)	0 − 30	5
Temperature (°C)	25 − 60	25
λ_max_ (nm)	400 − 600	450

^a^Solvents tested: methanol, ethanol, propanol, butanol, acetone, and acetonitrile.

### Molar ratio and mechanism of the reaction

**Figure 4:**
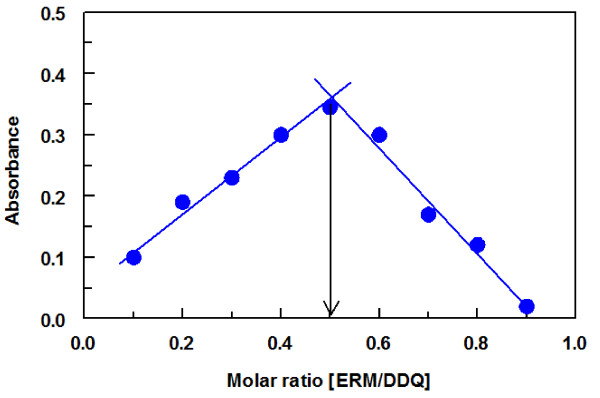
Job's plot for CT reaction of CLM with DDQ.

**Figure 5:**
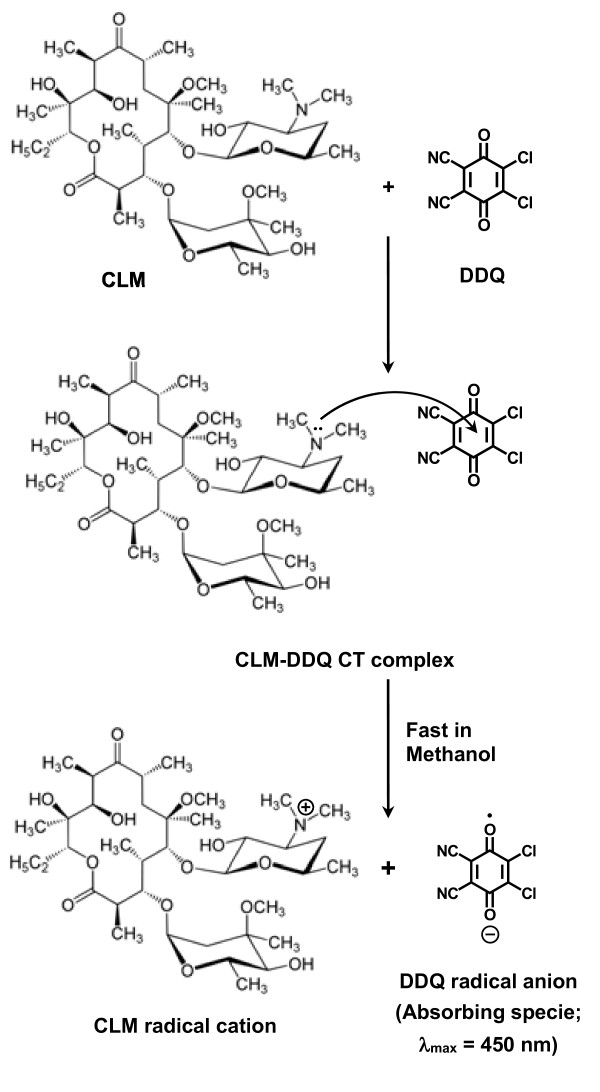
Mechanism of CT reaction of CLM with DDQ.

Job’s method of continuous variation [31] was used for determining the molar ratio of CLM to DDQ. From the obtained Job’s plot, it was concluded that the CLM:DDQ ratio is 1:1 (Figure 4). This indicated that one mole of DDQ interacted with one mole of CLM and confirmed the assumption rose before when the effect of DDQ concentration was studied (Figure 3). Considering the presence of one nitrogen atoms in CLM structure (dimethylamine group in the sugar moiety), the reaction was postulated to proceed as shown in Figure 5. One free electron of the nitrogen atom was transferred to the charge-deficient center of DDQ molecule.

### Validation of the proposed assay

#### Calibration, range, and sensitivity

Under the above mentioned optimum reaction conditions, the calibration curve for the analysis of CLM by the proposed assay was constructed by plotting the absorbances as a function of the corresponding CLM concentrations. The regression equation for the results was derived using the least-squares method Beer’s law plot (11-points) was linear in the range of 20–850 μg mL^−1^. The calibration equation was: A = 0.0073 + 0.0018 C (r = 0.9985), where A, C and r are the absorbance, concentration of CLM and correlation coefficient, respectively. The limits of detection (LOD) and quantitation (LOQ) were determined [34]. The following formula was used: LOD or LOQ= ×SDa/b, where × = 3.3 for LOD and 10 for LOQ, SDa is the standard deviation of the intercept, and b is the slope. The LOD and LOQ were found to be 15.5 and 51.2 μg mL^−1^, respectively. The quantitative parameters of the proposed assay are given in Table 2.

**Table 2:** Quantitative parameters for the analysis of CLM by the proposed assay

**Parameter**	**Value**
Range ( μg mL^−1^)	20 − 850
Intercept	0.0073
Slope	0.0018
Correlation coefficient	0.9985
LOD ( μg mL^−1^)	15.5
LOQ ( μg mL^−1^)	51.2

#### Accuracy and precision

Accuracy of the proposed assay was assessed by analytical recovery studies. Recovery was determined by the standard addition method. Known amounts of CLM were added to pre-determined drug-containing pharmaceutical formulation, and then determined by the proposed assay; 3 replicates were used in each experiment. The analytical recovery was calculated and found to be 97.3−101.5 ± 0.25−1.54%. This range is acceptable according to the guidelines for validation of analytical procedures [34], indicating the accuracy of the proposed assay.

The precisions of the proposed assay were determined on samples of drug solutions at three concentration levels for each drug (Table 3) by analyzing 5 replicates of each sample as a batch in a single assay run. The relative standard deviations (RSD = standard deviation/mean; expressed as percentage) did not exceed 1.82% (Table 3) proving high precision of the assay for the routine application in quality control laboratories. This high level of precision was attributed to the accuracy of the volumes that have been concomitantly dispensed in the microwells by multi-channel pipettes, and completeness of the reaction in a small volume (200 μL).

**Table 3:** Precision of the proposed assay at different CLM concentrations

**Concentration (μg mL**^**−1**^**)**	**Relative standard deviation**	
	**Within-assay, n = 5**	**Between-assays, n = 5**
50	1.25	1.82
250	0.98	1.05
500	1.34	1.65

#### Selectivity

The selectivity of the proposed methodology was assessed by studying the possible interferent species on the reaction of CLM with DDQ. Samples were prepared by mixing known amount (50 mg) of CLM with various amounts of the common excipients (starch, microcrystalline cellulose, silica gel, talc, titanium dioxide, and magnesium stearate) were tested. The results (Table 4) revealed that no interference was observed from any of these excipients with the proposed methodology as the average recovery value was 98.8 ± 1.65%. The absence of interference from these excipients was attributed to the extraction of CLM from the samples by methanol which does not dissolve these excipients.

**Table 4:** Analysis of CLM in the presence of common excipients by the proposed assay

**Excipient**	**Recovery (% ± SD)**^**a**^
Starch (50)^b^	97.9 ± 1.54
Microcrystalline cellulose (10)	100.2 ± 0.25
Silica gel (10)	98.5 ± 1.34
Talc (10)	97.4 ± 0.89
Titanium oxide (10)	101.3 ± 1.02
Magnesium stearate (10)	97.2 ± 1.54
Average ± SD	98.8 ± 1.65

^a^Values are mean of three determinations.

^b^Figures in parenthesis are the amounts in mg added per 50 mg of CLM.

The ability of the proposed method to detect intact CLM in presence of its degradation products and/or related substances, if any, was not studied in the methodology described herein. This was attributed to the common inherent disability of most CT-based spectrophotometric method, rather than the separation-based methods, to act as stability-indicating methods.

#### Robustness and ruggedness

Robustness was examined by evaluating the influence of small variation in the method variables on its analytical performance. In these experiments, one parameter was changed whereas the others were kept unchanged, and the recovery percentage was calculated each time. It was found that small variation in the method variables did not significantly affect the procedures; recovery values were 98.59 – 102.18 ± 0.58 – 1.24%. This indicated the reliability of the proposed methodology during its routine application for the analysis of CLM.

Ruggedness was also tested by applying the proposed methodology to the assay of CLM using the same operational conditions but using two different instruments at two different laboratories and different elapsed time. Results obtained from lab-to-lab and day-to-day variations were reproducible, as the relative standard deviations (RSD) did not exceed 2%.

### Application of the proposed assay

The commercially available pharmaceutical formulations of CLM (Klaricid® tablets) were subjected to the analysis by the proposed and validated methods [10] and the obtained results were then statistically compared with each other. The mean percentage recovery, relative to the labeled amounts, obtained by the proposed methodology was 99.5 ± 1.81 (Table 5). In the t- and F-tests, no significant differences were found between the calculated and theoretical values of both the proposed and the reported assays at 95% confidence level. This indicated similar accuracy and precision in the analysis by the proposed and reported methods.

**Table 5:** Analysis of CLM in tablets by the proposed and reported methods

**Tablet**	**Content (% ± SD)**^**a**^		**t-Value**^**b**^	**F-value**^**b**^
	**Proposed method**	**Reported method**		
Klaricid® tablets	99.5 ± 1.81	99.35 ± 1.12	0.62	2.61

^a^Values are mean of five determinations ± SD.

^b^The tabulated values at 95% confidence limit are 2.78 and 6.39, respectively.

## Conclusions

The present study described the development and validation of a novel assay for the CT-based spectrophotometric determination of CLM. In this assay, the reaction was carried out in 96-microwell plates and the color signals were measured by microwell-plate reader. The assay was fully validated according to the guidelines for validation of analytical procedures and the results were satisfactory. The applicability of the proposed methodology for the quality control of CLM was confirmed as indicated by the acceptable recovery values. The assay described herein has the following advantages:

– Providing a high throughput analysis that enables the processing of large number of samples in a short time. This property was attributed to the use of multi-channel pipettes for efficient dispensing the solutions, carrying out the analytical reaction in 96-well plates (as reaction vessels), and measuring the color signals in the 96 wells at ~ 30 seconds by the plate reader.

– Reducing the consumption of organic solvents in the CT-based spectrophotometric analysis of CLM, accordingly reduction in the exposures of the analysts to the toxic effects of organic solvents.

– Reduction in the analysis cost by 50-folds (200 μL in the present methodology versus 10,000 μL in the reported conventional CT-based spectrophotometric methods [24,25]) which can be reflected on the price for the finished dosage forms, thus it can reduce the expenses for the medications to the patients.

## Abbreviations

CLM: Clarithromycin; CT: Charge-transfer; DDQ: 2,3-dichloro-5,6-dicyano-1,4-benzoquinone; LOD: Limit of detection; LOQ: Limit of quantification; SD: Standard deviation; RSD: Relative standard deviation.

## Competing interests

The authors declare that they have no competing interests.

## Authors’ contributions

IAD suggested the subject of the study, designed the study, participated in results discussion, and revised the manuscript. MAA conducted the optimization of reaction variables and assay validation studies. TAW conducted the application of the assay and participated in preparing the manuscript. All authors have read and approved the final manuscript.
